# Activation of human brown adipocytes alters the plasma fatty acid profile

**DOI:** 10.3389/fmolb.2026.1842960

**Published:** 2026-08-26

**Authors:** Katarzyna Miniewska, Patrycja Mojsak, Sandra Chmielewska, Paulina Choinska, Joanna Godzien, Lukasz Labieniec, Andrzej Andrejczuk, Jarna Hannukainen, Vesa Oikonen, Kirsi Virtanen, Pirjo Nuutila, Michal Ciborowski, Katarzyna Maliszewska, Adam J. Kretowski

**Affiliations:** 1 Department of Medical Biochemistry, Medical University of Bialystok, Bialystok, Poland; 2 Metabolomics and Proteomics Laboratory, Clinical Research Centre, Medical University of Bialystok, Bialystok, Poland; 3 Faculty of Physics, University of Bialystok, Bialystok, Poland; 4 Turku PET Centre, Turku, Finland; 5 Department of Endocrinology, Diabetology and Internal Medicine, Medical University of Bialystok, Bialystok, Poland

**Keywords:** brown adipose tissue, cold exposure, fatty acids, gas chromatography-mass spectrometry, human, lipid metabolism, metabolomics

## Abstract

**Introduction:**

Even though brown adipose tissue (BAT) has recently been identified as a possible target for combating obesity and type 2 diabetes, neither the mechanisms of its activation nor its relationships to clinical parameters are clearly defined. Metabolomics enables the simultaneous analysis of multiple classes of compounds, thus seems to be a perfect tool to study this topic. This study aimed to explore alterations during cold exposure (CE) associated with the activation of BAT.

**Materials and methods:**

Twenty-five healthy male adults underwent 2 hours of CE followed by PET-MRI. Afterwards, they were divided into two groups, depending on BAT presence (BATpos or BATneg). The levels of metabolites were measured in plasma samples at three time points: before, after one, and 2 hours of CE, using a standard gas chromatography-mass spectrometry (GC-MS) method for metabolite profiling.

**Results:**

In the BATpos group, the levels of linoleic, oleic, and palmitic acids were significantly increased after 120 min of CE compared to the baseline. These changes were absent in the BATneg group. When comparing the BATpos and BATneg groups, we observed that the BATpos group had significantly lower baseline level of succinic acid. After 60 min of CE, the BATpos group showed significantly increased levels of linoleic, oleic and trans-13-octadecenoic acid. The differences in oleic acid persisted for another hour.

**Conclusion:**

Between-group differences in the level of fatty acids during CE, along with the BATpos group-specific alterations during CE, suggest that the activation of BAT affects lipid metabolism.

## 1 Introduction

Obesity-related diseases, such as diabetes, hyperlipidemia or different types of cancer (pancreatic, colon or endometrial) are still an emerging public health problem; therefore, it is essential to seek new therapeutic tools to fight obesity (Grundy, 2016; Caballero, 2019). Although losing weight can be achieved through the implementation of dietary changes and physical activity, maintaining lifestyle modifications may be challenging, and weight regain is likely to occur (Ochner et al., 2013). With the development of GLP-1 and GIP/GLP-1 receptor agonists, obesity treatment may become easier and more effective. However, side effects (mainly gastrointestinal adverse events) may cause patients to discontinue the treatment. Moreover, there are individuals described as non-responders, characterized by a reduction in body weight of less than 5% (Garvey et al., 2022; Rubino et al., 2021; Wilding et al., 2021; Wadden et al., 2021).

Brown adipose tissue (BAT) appears to be a promising target because it utilizes its own fat depots to generate heat through thermogenesis. This process is driven by β3-adrenergic stimulation due to cold exposure (CE), leading to increased energy expenditure and potentially improving the metabolic state. It has been shown that BAT activation leads to an increase in energy expenditure, improved glucose and lipid homeostasis, and a decrease in body fat content. Furthermore, it diminishes insulin resistance (Cheng et al., 2021; Chondronikola et al., 2016). Thermogenesis in BAT is possible thanks to numerous mitochondria and uncoupling protein 1 (UCP1), located in their inner membrane (Sun et al., 2025). The heat production in BAT relies on fatty acids (FAs) generated from the lipolytic degradation of triacylglycerols (TGs) not only in BAT but also in white adipose tissue (WAT) (Shin et al., 2017; Schreiber et al., 2017). Glucose is also an important substrate in BAT metabolism but it is mostly used for lactate and TGs synthesis. It supports the production of fatty acids that are subsequently esterified to TGs which are simultaneously broken down to drive thermogenesis (Carpentier et al., 2023).

BAT may be activated by several factors other than CE, including pharmacological and dietary interventions (Hachemi and U-Din, 2023; Negroiu et al., 2024). β-adrenergic receptor agonists, such as mirabegron, have been studied for their ability to stimulate non-shivering thermogenesis; however, recent findings suggest that β2-adrenergic signaling might be more relevant than β3-adrenergic pathways. Certain dietary compounds, like omega-3 fatty acids, capsaicinoids from chili peppers and green tea catechins may also activate BAT through sympathetic activation, thermogenic gene expression and metabolic regulation. The data on the influence of physical exercise on BAT stimulation are ambiguous: some studies suggest an increase, while other a decrease in BAT activity.

Metabolomics is a comprehensive approach that focuses on the analysis of small compounds, thereby enabling the understanding of the mechanisms of action of various biological processes, as well as identifying disease biomarkers (Pardali et al., 2025). With this approach, it is possible to determine the levels of numerous compounds from different classes during a single analysis. The most common technique used in metabolomics research is mass spectrometry (MS), usually coupled with chromatography, either liquid or gas (LC or GC) (Castro-Alves et al., 2025). As thermogenesis is strongly dependent on FAs, GC-MS, with its particular utility for the analysis of this class of molecules, seems to be the best choice for studying metabolome changes due to CE.

Consequently, the present study aimed to investigate the influence of CE and BAT presence (assessed based on the glucose uptake measured with the use of PET/MRI) on FA profile measured with GC-MS in human plasma samples. Another objective was to analyze the associations between FAs and endocannabinoids (ECs) dynamics during CE.

## 2 Materials and methods

### 2.1 Study group

The CE procedure and positron emission tomography-magnetic resonance imaging (PET-MRI) were performed on a group of 37 healthy adult males between October 2016 and April 2018 (only in the autumn and winter). Out of the whole group, 17 individuals had active BAT (the study group). The control group was matched for BMI and age to the study group. Eventually, the group consisted of 25 healthy, non-smoking Caucasian males (n = 17 in the study group, hereinafter referred to as BATpos; n = 8 in the control group, BATneg). The same cohort was described in detail in our previous paper, where demographic and clinical characteristics were reported (Miniewska et al., 2022). Briefly, neither differences in glucose or insulin level during a 120-min oral glucose tolerance test (OGTT) nor lipid profile were observed.

### 2.2 Screening visit, cold exposure and PET-MRI

**FIGURE 1:**
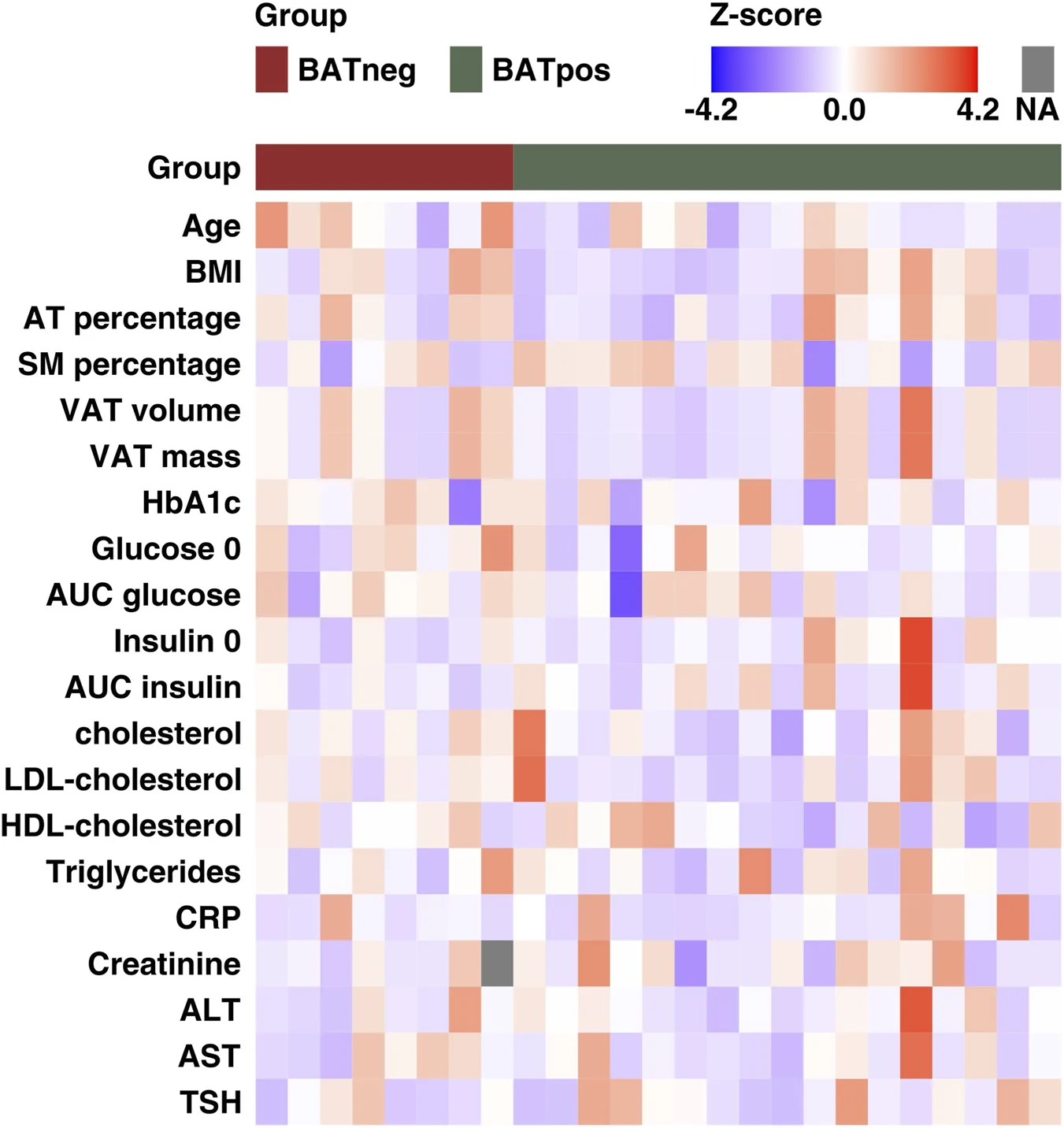
Z-score heatmap visualisation of BATneg and BATpos groups. BMI–body mass index; AT percentage–percentage of adipose tissue in total body mass; SM percentage–percentage of skeletal muscles in total body mass; VAT–visceral adipose tissue; HbA1c–glycated haemoglobin; Glucose 0 – fasting glucose concentration; Insulin 0 – fasting insulin level; AUC glucose–area under the curve of glucose during oral glucose tolerance test; AUC insulin–area under the curve of insulin during oral glucose tolerance test; LDL–low density lipoprotein; HDL–high density lipoprotein; CRP–C-reactive protein; ALT–alanine transaminase; AST–aspartate transaminase; TSH–thyrotropic hormone.

During the screening visit, participants arrived at the medical facility in a fasting state for laboratory tests and body composition analysis (Figure 1; Supplementary Table S1). To assess glucose metabolism, a 75 g OGTT was performed according to WHO guidelines. Blood was collected for glucose and insulin measurement (in plasma or serum, respectively) before and 30, 60 and 120 min after glucose ingestion. For glucose concentration measurement, the enzymatic hexokinase method was applied (Roche Diagnostics, Basel, Switzerland). For insulin level measurement, the immunoradiometric assay (DIAsource, Nivelles, Belgium) was used. During the same visit, body composition analysis was performed. The two following methods were applied: bioimpedance (InBody 720, Biospace, Seoul, Korea) and dual energy X-ray absorption (DXA) (Lunar iDXA, GE Healthcare, Chicago, IL, USA) to assess the following parameters: adipose tissue percentage, skeletal muscle percentage, visceral adipose tissue (VAT) volume and mass.

Participants who met the inclusion criteria underwent CE followed by PET-MRI. To activate BAT, a personalized procedure of CE was applied, during which blood was collected for further analyses (before and after 60 and 120 min of CE (Miniewska et al., 2022; Maliszewska et al., 2022; Supplementary Figure S1). The activity of BAT was assessed based on the glucose uptake during PET-MRI (Virtanen et al., 2001). BAT was considered activated when the glucose uptake rate was higher than 2.0 µmol/(100 g × min). Based on this examination, the individuals were assigned to the BATpos group (with a median BAT activity of 29.9 µmol/(100 g × min) and a BAT volume of 15,280 mm^3^) or the BATneg group. The details about CE procedure, PET-MRI and BAT assessment are provided in Supplementary Material.

### 2.3 GC-MS analysis, data treatment and statistical analysis

Untargeted GC-MS-based metabolomics analysis was performed on EDTA-plasma samples collected during CE. Plasma samples were obtained by the centrifugation of whole blood and stored in Eppendorf tubes at −80 °C until the day of analysis. Samples were prepared and analyzed according to a previously described method (Mojsak et al., 2022). The description of the protocol is provided in Supplementary Material.

Following the analysis, variables were filtered as proposed by Godzien et al. (Godzien et al., 2015). Missing values were replaced by k-means nearest neighbor (Armitage et al., 2015) using imputomics–web server and R package for missing values imputation in metabolomics data (Chilimoniuk et al., 2024).

Sample areas were normalized by internal standard abundance (4-nitrobenzoic acid) to minimize the response variability of the instrument. Data were filtered based on the coefficient of signal variation (CV) in quality control samples with the threshold of 30% considered as acceptable.

Statistical analysis was performed using R software (version 4.4.2, https://www.R-project.org/, accessed on 5 June 2025). For paired comparisons, the Wilcoxon test was used, whereas for non-paired comparisons, the Mann-Whitney test was employed. The associations between metabolites’ levels and other continuous variables were calculated using Spearman’s correlation analysis.

Non-parametric methods were chosen due to the relatively small sample size.

### 2.4 Ethics

The study protocol conformed to the principles of the Declaration of Helsinki and was approved by the Ethics Committee of the Medical University of Bialystok (R-I-002/233/2015, R-I-002/203/2019, APK.002.129.2024). All participants provided their written informed consent.

## 3 Results

**FIGURE 2:**
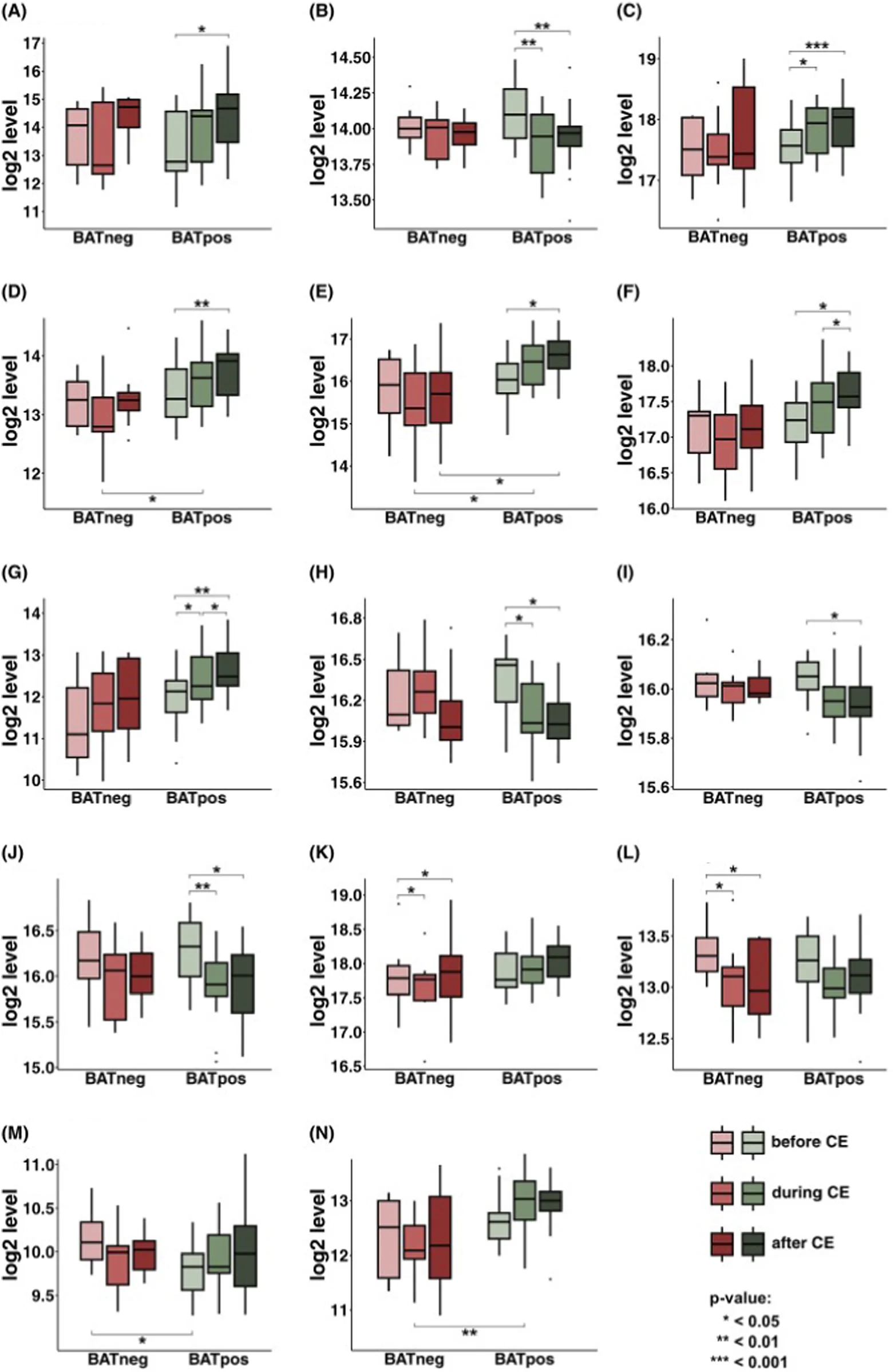
Levels (log2 transformed) of metabolites altered during CE and/or differentiating BATneg and BATpos groups. CE – cold exposure. **(A)** citric acid; **(B)** glyoxylic acid; **(C)** glycerol; **(D)** linoleic acid; **(E)** oleic acid; **(F)** palmitic acid; **(G)** palmitoleic acid; **(H)** N-methylacetamide; **(I)** N-Methyltrifluoroacetamide; **(J)** pyruvic acid; **(K)** 2-ketoisocaproic acid; **(L)** 3-methyl-2-oxobutanoic acid; **(M)** succinic acid; **(N)** trans-13-octadecenoic acid.

We evaluated the alterations in plasma metabolic profile by the use of GC-MS. We noticed that BATpos individuals showed an increase of citric acid (p = 0.01), glyoxylic acid (p = 0.008), glycerol (p < 0.001), linoleic acid (p = 0.006), oleic acid (p = 0.01), palmitic acid (p = 0.01), palmitoleic acid (p = 0.002), and a decrease of N-methylacetamide (p = 0.01), N-Methyltrifluoroacetamide (p = 0.01), pyruvic acid (p = 0.01) in 120 min versus the beginning of CE (Figures 2A–J). We did not observe those changes in participants without BAT. However, in the BATneg group, the 2-ketoisocaproic acid and 3-methyl-2-oxobutanoic acid decreased after 60 min (p = 0.02 and p = 0.02, respectively) and 120 min (p = 0.02 and p = 0.04, respectively) of CE versus baseline values (Figures 2K,L). P-values are presented in Table 1.

**TABLE 1:** Changes in metabolites during CE in BATpos and BATneg groups, and between-group comparisons at each time point. P-values for within-group comparisons during CE were calculated using Wilcoxon test; p-values for between-group comparisons were calculated using Mann-Whitney U test. A p-value <0.05 was considered statistically significant. NS–not significant.

Metabolite	Changes during CE				Changes between BATpos and BATneg groups		
	BATpos		BATneg				
	60′ vs. 0′	120′ vs. 0′	60′ vs. 0′	120′ vs. 0′	0′	60′	120′
citric acid	0.01	NS	NS	NS	NS	NS	NS
glyoxylic acid	0.008	0.008	NS	NS	NS	NS	NS
glycerol	0.01	<0.001	NS	NS	NS	NS	NS
linoleic acid	NS	0.006	NS	NS	NS	0.02	NS
oleic acid	NS	0.01	NS	NS	NS	0.03	0.04
palmitic acid	NS	0.02	NS	NS	NS	NS	NS
palmitoleic acid	0.03	0.002	NS	NS	NS	NS	NS
N-methylacetamide	0.02	0.01	NS	NS	NS	NS	NS
N-Methyltrifluoroacetamide	NS	0.02	NS	NS	NS	NS	NS
pyruvic acid	0.003	0.01	NS	NS	NS	NS	NS
2-ketoisocaproic acid	NS	NS	0.02	0.02	NS	NS	NS
3-methyl-2-oxobutanoic acid	NS	NS	0.02	0.04	NS	NS	NS
succinic acid	NS	NS	NS	NS	0.02	NS	NS
trans-13-octadecenoic acid	NS	NS	NS	NS	NS	0.009	NS

Moreover, when we compared both BATpos and BATneg groups in terms of changes in plasma metabolome before (0 min), during (60 min) and after (120 min) of CE, we noticed that before CE groups differentiated in the concentration of succinic acid (p = 0.02) (Figure 2M). However, in 60 min of BAT activation, groups varied in the levels of linoleic acid (p = 0.02) and trans-13-octadecenoic acid (p = 0.009) (Figures 2D,N). Moreover, the concentration of oleic acid distinguished BATpos and BATneg groups, both during and after CE (Figure 2E). P-values are presented in Table 1.

**FIGURE 3:**
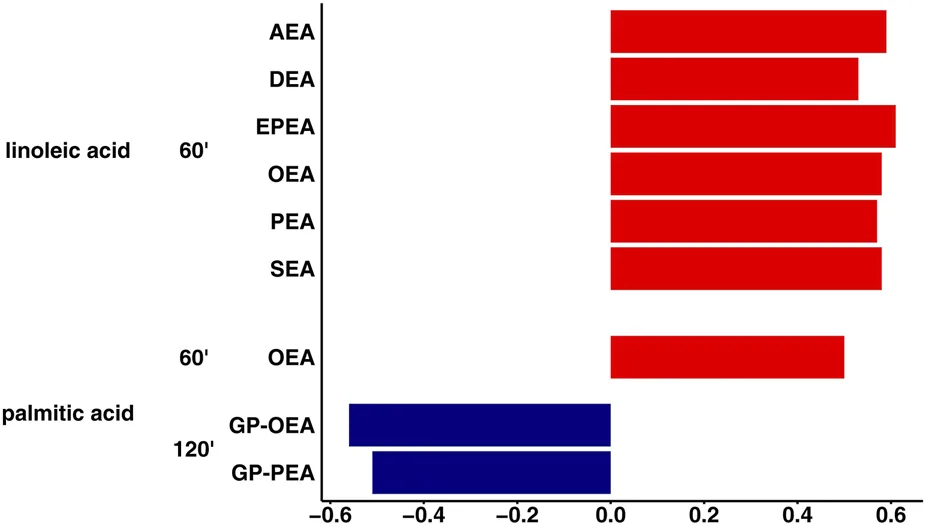
Correlation coefficients in BATpos group between fatty acids and eCBs. AEA–arachidonoylethanolamide; DEA–docosatetraenoylethanolamide; EPEA–eicosapentaenoyl ethanolamide; GP-OEA–glycerophospho-N- oleoylethanolamide; GP-PEA–glycerophospho-N- palmitoylethanolamide; OEA–oleoylethanolamide; PEA–palmitoylethanolamide; SEA–stearoylethanolamide.

In the previously published paper, where study and control groups were the same as in the presented study, we noticed the relationship between BAT presence and endocannabinoid (eCB) profile, i.e. stearoylethanolamide (SEA), palmitoylethanolamide (PEA), oleoylethanolamide (OEA), eicosapentaenoyl ethanolamide (EPEA), docosatetraenoylethanolamide (DEA), arachidonoylethanolamide (AEA), glycerophospho-N- oleoylethanolamide (GP-OEA) and glycerophospho-N-palmitoylethanolamide (GP-PEA) (Mal et al., 2023). In this paper, we included those results and evaluated the statistical significance between the eCB profile with the outcomes from the FA profile assessed by GC-MS (Figure 3). We found that in the BATpos group, the linoleic acid in 120 min of CE positively correlated with the following eCBs: SEA (p = 0.02, r = 0.58), PEA (p = 0.02, r = 0.57), OEA (p = 0.02, r = 0.58), EPEA (p = 0.01, r = 0.61), DEA (p = 0.03, r = 0.53) and AEA (p = 0.01, r = 0.59). Moreover, the presence of BAT was associated with a positive correlation between palmitic acid and OEA in 60 min (p = 0.04, r = 0.50), and a negative correlation between palmitic acid and GP-OEA and GP-PEA in 120 min of CE (p = 0.02, r = −0.56; p = 0.04, r = −0.51, respectively).

## 4 Discussion

BAT is considered a potential factor in lipid homeostasis, as it plays a role in lipid clearance in humans (Ouellet et al., 2011) by utilizing glucose and triglyceride for thermogenesis. Moreover, it increases whole-body lipolysis, triglyceride-free fatty acid (FFA) cycling, FFA oxidation and enhances adipose tissue insulin sensitivity (Chondronikola et al., 2016). Additionally, BAT is recognized as a secretory organ with a relevant local and systemic action (Villarroya et al., 2017).

The new possibilities for lipid analyses, such as GC-MS, present a powerful tool for the identification of new molecules associated with the activation of BAT.

Our results are consistent with the previously published papers. Kulterer et al. showed that in humans, brown adipocytes were associated with an anti-inflammatory PUFA profile as well as cytochrome P450 products. However, reduced concentrations of some pro-inflammatory hydroxyeicosatetraenoic acids were observed when compared to participants without detectable BAT (Kulterer et al., 2020). In our study, we also noticed that the fluctuation in oleic acid level was maintained the longest in the BATpos group during CE. It is the most common MUFA in human adipose tissue, with its beneficial effects on the lipid profile, blood pressure, and weight loss (Tutunchi et al., 2020). Additionally, another polyunsaturated omega-6 FA detected in our BATpos group was linoleic acid, known for lowering cardiovascular risk (Marklund et al., 2019). Succinic acid was shown to differentiate between the BATpos and BATneg groups even before CE in our study. It is a respiratory substrate of the mitochondrial electron transport chain and is involved in ATP formation, exhibiting a very high affinity to BAT (Jung et al., 2022). Moreover, succinic acid induces uncoupling protein 1-dependent thermogenesis, which helps reduce diet-induced obesity and enhance glucose tolerance (Mills et al., 2018). BAT is recognized as a metabolically healthy fat, as it increases energy expenditure and combats obesity and its complications (Maliszewska and Kretowski, 2021). Those alterations in lipid profiles may support the explanation of the mechanism through which brown adipocytes might relieve obesity, but they could also create a new therapeutic possibility. The results obtained in an isolated system indicate that long-chain FAs act as a physiological trigger of the UCP in BAT (Strieleman et al., 1985). Moreover, as shown by Armstrong et al., treatment with oleic acid, compared to palmitic acid, stimulated the expression of UCP-2, a homolog of the BAT uncoupling protein, in the hepatocyte, which may support the hypothesis of UCP-2 involvement in regulating energy metabolism (Armstrong and Towle 2001). Palmitic, oleic, and linoleic acids presented in TGs of BAT are released as a result of cyclic AMP activation of lipolysis. They may regulate BAT metabolism by self-controlling lipolysis and by acting as regulators of mitochondrial oxygen consumption in BAT (Bukowiecki et al., 1981).

What is important to notice is the fact that the increase in circulating free fatty acids during CE may not exclusively reflect BAT activity. Cold-induced sympathetic nervous system activation stimulates lipolysis in WAT through β-adrenergic signaling, resulting in the release of FAs and glycerol into the circulation. Zhang et al. demonstrated that CE activates innervation of WAT, leading to enhanced lipolysis and the release of FAs that support adaptive thermogenesis (Zhang et al., 2024). Furthermore, Chondronikola et al. showed that WAT and BAT are functionally connected through neural circuits: lipolysis in WAT promotes sympathetic drive to BAT and stimulates BAT thermogenesis (Chondronikola et al., 2016). Garrettson et al. demonstrated that β3-adrenergic stimulation of WAT induces local lipolysis and promotes BAT thermogenesis, supporting the concept of coordinated WAT-BAT response to adrenergic stimulation (Garretson et al., 2016). Moreover, Mottillo et al. suggested that chronic β3-adrenergic activation markedly increased lipid turnover, TG/FFA cycling, and *de novo* lipogenesis in both WAT and BAT, indicating that systemic adrenergic activation induces extensive metabolic remodeling in different types of adipose tissues, not only in BAT (Mottillo et al., 2014). Notably, FAs released during stimulated WAT lipolysis, including oleic, palmitic, linoleic, arachidonic, and eicosapentaenoic acids, were implicated as substrates and signaling molecules that support thermogenic activation. Therefore, the increased levels of circulating FAs observed in BATpos individuals in our study may reflect not only direct BAT activation but also enhanced sympathetic activation of WAT and increased whole-body lipid mobilization. Taken together, these findings suggest that the plasma lipid profile observed during CE likely reflects the integrated response of both WAT and BAT to sympathetic stimulation. However, the distinct lipid alterations observed in BATpos group indicate that BAT contributes to this metabolic adaptation.

Evidence from previous studies further supports that BAT activation is accompanied by remodeling of systemic lipid metabolism during CE. Iwen et al. showed that acute CE in healthy men increased insulin sensitivity and induced changes in circulating FAs, including very-long-chain saturated FAs and long-chain MUFAs, suggesting preferential uptake and use of specific lipid substrates by BAT (Iwen et al., 2017). Likewise, Straat et al. demonstrated that CE dynamically alters plasma lipidome, initially decreasing shorter and more saturated TGs while increasing longer-chain PUFA-enriched TGs and circulating FFAs (Straat et al., 2022). Their mechanistic studies suggested that enhanced WAT lipolysis and hepatic lipid remodeling provide energy substrates required for non-shivering thermogenesis. These findings are consistent with our results, in which BATpos individuals exhibited increases in linoleic acid, oleic acid and palmitic acid during CE, whereas such changes were not observed in BATneg group. In particular, the sustained elevation of linoleic and oleic acid in BATpos group supports the concept that BAT activation is associated with mobilization of PUFAs and MUFAs. Importantly, while studies by Iwen et al. as well as by Straat et al. compared lipid profiles before and after CE, our results show that the presence of BAT is linked to a distinct metabolic response to CE.

Considering the broader metabolic context, BAT is known to alleviate the chronic inflammatory statem linked with obesity. PUFAs have an anti-inflammatory effect on obesity through the regulation of lipid metabolism and epigenetic modulation (Herna et al., 2015). This paper demonstrates a relationship between plasma lipid profile and BAT presence. However, we have previously evaluated the influence of diet, macronutrients, and FA intake on BAT activation, suggesting that BAT activity might be related to daily omega-6 FA intake (Maliszewska et al., 2022).

In the present study, we detected the alteration in a saturated FA (palmitic acid) independently of PUFAs and MUFAs. Despite less favorable effects of palmitic acid on human health, it is assumed that not only their presence, but their ratio is crucial to maintain a metabolically beneficial profile (Carta et al., 2017).

Clinical studies investigating CE have similarly reported improvements in lipid profiles and cardiometabolic markers, supporting our observations. Similarly to our study, Jurado-Fasoli et al. also showed that the human plasma lipid profile of study participants who underwent cooling was healthier from a cardiometabolic perspective (Jurado-Fasoli et al., 2024). Cold-induced changes in PUFAs were inversely related to adiposity, glucose homeostasis, lipid profile, and liver parameters, and were slightly correlated with BAT volume. Moreover, the cold-induced activation of BAT was not only associated with the release of PUFAs but also with changes in the eCBs, which is consistent with our previously published data (Maliszewska et al., 2023).

In our study, we also observed that a lack of brown adipocytes was associated with the decrease of plasma 2-ketoisocaproic acid and 3-methyl-2-oxobutanoic acid during participants’ cooling. In turn, 2-ketoisocaproic acid is a metabolic intermediate in the biochemical pathway of leucine, which is responsible for skeletal muscle protein synthesis, regeneration and proteolysis (Wilson et al., 2013). At the same time, α-ketoisocaproic acid is converted into β-hydroxy-β-methyl butyrate by a branched-chain amino acid in skeletal muscles (Osuka et al., 2021). Furthermore, β-hydroxy-β-methyl butyrate controls lipid metabolism, increases insulin sensitivity (Duan et al., 2023), activates adaptive thermogenesis and induces browning of WAT (Yang et al., 2025). The relationship between muscle mass and BAT was assessed previously; in our published paper, we showed a direct correlation between BAT and increased muscle mass and lower visceral fat tissue accumulation (Maliszewska et al., 2022).

Encouraged by the results of the current study and the findings of our prior research (Maliszewska et al., 2023), which showed alterations in plasma eCBs in the presence of brown adipocytes, we decided to explore the topic of mutual correlations between lipids and eCBs, depending on the presence of BAT. Our hypotheses were supported by clinical evidence pointing to the fact that the eCB system is regulated by PUFAs (Komarnytsky et al., 2021). In the present study, BATpos individuals have been characterized by an increased level of linoleic acid, among others, which is one of the two essential dietary PUFAs. Linoleic acid serves as a starting metabolite for the synthesis of omega-6 FAs (Burdge and Calder, 2005). Then they contribute to the synthesis of arachidonic acid, a precursor for primary endogenous cannabinoid receptor agonists, AEA and 2-arachidonoylglycerol (2-AG). In our study, we found that in the BATpos group, the linoleic acid level at 120 min of CE positively correlated with AEA. Krott et al. (Krott et al., 2016) reported results consistent with our findings, showing a strong association between the eCB system and the control of BAT and WAT function mediated by the sympathetic nervous system. Activated brown adipocytes expressed a higher level of eCBs; moreover, β3-adrenoceptor activation caused browning of white adipocytes. For the first time, the authors demonstrated a potential role of CB1 receptors and their endogenous ligands in regulating energy homeostasis induced by CE and subsequent β3-adrenoceptor activation. Moreover, an intervention study in mice models with administration of rimonabant (the cannabinoid CB1 receptor antagonist) revealed that the long-term weight loss was at least partially caused by an increase in energy expenditure, manifested with higher temperature noted in BAT, mainly mediated by the central eCB system (Verty et al., 2009).

The major limitation of our study is the relatively small sample size, which is due to the high costs of PET−MRI and tracer purchase. Because of that, it is necessary to realize that our results show associations rather than prove biological mechanisms. In addition, because only healthy male participants were included, the findings cannot be directly generalized to women or broader populations. Our results ought to be further verified in a larger population and different ethnic groups. What is also essential to bear in mind is that the main source of energy for BAT are FAs (Blondin et al., 2017); therefore, it would be beneficial to use a tracer other than 18-FDG. We chose 18-FDG as a tracer for our study because it is more easily accessible. We may overlook the possibility that some subjects could be BATpos with a significant FA uptake by the BAT tissue, but low glucose uptake. By using 18-FDG, they would be categorized as BAT-negative. Moreover, FDG allow for the detection and localization of BAT, but it may be less precise in terms of BAT activity (Leitner et al., 2017). Another limitation of BAT is that brown adipocytes are interspersed within white adipose tissue. Thus, during PET-MRI, areas considered as BAT could contain both brown and some white adipocytes. Potentially, the CE procedure was not optimal for some subjects, mainly due to an increased amount of subcutaneous fat tissue, which may have led to false-negative results for BAT activity.

## 5 Conclusion

We demonstrated that the presence of BAT is associated with a specific lipid profile, as assessed by GC-MS. The increase of PUFAs, MUFAs and some saturated acids was observed in subjects with confirmed BAT presence. This finding supports the hypothesis that brown adipocytes may influence the metabolic process and alleviate obesity through their role in lipid metabolism and anti-inflammatory effects.

## Data Availability

The raw data supporting the conclusions of this article will be made available by the authors, without undue reservation.
