# Supplementary material for: Activation of human brown adipocytes alters the plasma fatty acid profile
Source: Front Mol Biosci. 2026 Aug 26;13:1842960. doi: 10.3389/fmolb.2026.1842960 (PMC13553035; doi:10.3389/fmolb.2026.1842960)
Supplement: Supplementary file 1 [file Supplementaryfile1.docx]

Supplementary Material

# 1 Cold exposure and PET-MRI

Participants who met the inclusion criteria underwent CE followed by PET-MRI. A personalised procedure of cooling was applied to activate BAT. Subjects lay on a bed between two water-filled blankets (Blanketroll III, Cincinnati Sub-Zero, Cincinnati, OH, USA). Initially, the water temperature was 22°C in order to acclimatized subjects for one hour. Subsequently, it was decreased by 2°C every 10 minutes until individuals either reported or showed signs of shivering. Blood was collected for further analyses before and after 60 and 120 minutes of CE. Next, a whole-body PET-MRI (Biograph mMR 3T, Siemens Healthcare, Erlangen, Germany) with an 18F-fluorodeoxyglucose injection (18F-FDG) (4 MBq/kg of body mass) was performed. The results were analysed using the Carimas software, developed at the Turku PET Centre in Finland. The activity of BAT was assessed based on the glucose uptake, which was calculated as follows: plasma glucose concentration × Ki × LC 1, where Ki stands for the influx rate constant of 18F-FGD, determined using the Gjedde-Patlak model and LC for the lumped constant (LC = 1.14 for adipose tissue). BAT was considered activated when the glucose uptake rate was higher than 2.0 μmol/(100g×min). Based on this examination, the individuals were assigned to the BATpos group (with a median BAT activity of 29.9 μmol/(100g×min) and a BAT volume of 15,280 mm³) or the BATneg group.

# 2 GC-MS analysis and data treatment

O-methoxyamine hydrochloride, heptane of analytical grade and pyridine were supplied from Sigma-Aldrich (Steinheim, Germany). N-Methyl-N-(trimethylsilyl)trifluoroacetamide (MSTFA) with 1% trimethylchlorosilane (TMCS) solution and acetonitrile (HPLC grade) were purchased from Thermo Fisher Scientific (Waltham, MA, USA).

Stearic acid methyl ester (C18:0 methyl ester) and 4-nitrobenzoic acid (4-NBA) were obtained from Sigma-Aldrich (Steinheim, Germany) and used as internal standards (ISs). The solution of 4-NBA (IS1) was prepared in methanol:water (v:v, 1:1), whereas the solution of methyl stearate was prepared in heptane (IS2). Two mixtures of standards for GC-MS, one containing grain fatty acid methyl esters (FAMEs) (C8:0-C22:1, n9) and the other with a mixture of n-alkanes (C8:C40), were obtained from Supelco (Bellefonte, PA, United States).

Untargeted GC-MS-based metabolomics analysis was performed on EDTA-plasma samples collected during CE. Plasma samples were obtained by the centrifugation of whole blood and stored in Eppendorf tubes at -80°C until the day of analysis. An aliquot of 50 µL plasma and 150 µL cold methanol:water (v:v, 1:1) containing the IS1 (25 ppm) was used for metabolite extraction. The mixture was vortexed for 2 minutes and centrifuged at 15,000 g for 10 minutes at 4°C. Finally, the supernatant obtained from each sample (150 µL) was collected into a GC vial equipped with an insert and was evaporated to complete dryness using a vacuum concentrator.

Next, all samples were subjected to a two-step derivatisation process. First, methoxymation was performed by adding 30 µL of methoxylamine hydrochloride in pyridine solution (30 mg/mL). The samples were then incubated at room temperature in the dark for 16 hours. The second step was silylation, with the addition of 30 µL of MSTFA + 1% TMCS to each sample. Subsequently, samples were placed in the oven to react for 1 h at 70 °C. Eventually, 60 µL of heptane containing IS2 (10 ppm) was added. Metabolites were analysed using the GC-MS system described below.

The blank samples containing cold methanol:water (v:v, 1:1) were used to detect the contamination of the column as well as the background noise produced during sample derivatisation, data processing and GC-MS analysis. The quality control (QC) samples were used to monitor the analytical variability and to assess the reproducibility and repeatability of the method (16). The QC samples were prepared by pooling an equal volume of all the study samples.

Metabolic fingerprinting was conducted using a GC system (series 7890B) equipped with a 7693A auto-sampler and a Mass Selective Detector 7000D (Agilent Technologies, Palo Alto, CA, USA). One µL of the derivatised sample with ISs was injected into a DB–5MS capillary GC column (30 m × 0.25 mm × 0.25 µm) using helium as the carrier gas at a constant gas flow of 1.0 mL/min. The injector temperature was set to 250°C, and the split ratio to 1:10. The temperature gradient program was initiated at 60°C and was held for 1 min, followed by a subsequent increase in temperature to 320°C at a rate of 10°C/min. The GC-MS transfer line, filament source and quadrupole temperature were set to 280, 230, and 150°C, respectively. The electron ionisation source was set to 70 eV, and the mass spectrometer was operated in full scan mode, with a mass range from m/z 50 to 500 at a scan rate of 1.38 scan/s.

The deconvolution and identification were performed using Mass Hunter Quantitative Unknowns Analysis software (B.07.00, Agilent, Santa Clara, CA, USA), alignment with Mass Profiler Professional software (version 13.0, Agilent, Santa Clara, CA, USA) and peak integration with Mass Hunter Quantitative Analysis software (version B.07.00, Agilent, Santa Clara, CA, USA). The identification was primarily based on accurate mass and product ion spectrum matching against an in-house library of authentic standards, as well as Fiehn’s and NIST 14 libraries.

Figure S1. Cold exposure and PET-MRI scheme.

Table S1. Characteristics of BAT (+) and BAT (-) group.

| Parameter | | BATpos  median (Q1 – Q3) | BATneg  median (Q1 – Q3) | p-value |
| --- | --- | --- | --- | --- |
| **N** | | 17 | 8 |  |
| **BAT activity**  **[µmol × (100 g^-1^) × min^-1^]** | | 29.92 (23.62 – 37.75) | NA |  |
| **BAT volume [mm^3^]** | | 15280 (1859 – 31490) | NA |  |
| **Age [years]** | | 24 (23 – 26.5) | 27 (25 – 32.25) | 0.07 |
| **BMI [kg/m^2^]** | | 24.48 (22.61 – 28.71) | 26.73 (23.17 – 31.07) | 0.37 |
| **% AT [%]** | | 16.5 (13.25 – 21.95) | 21.95 (16.03 – 25.88) | 0.34 |
| **% SMM [%]** | | 47.54 (44.23 – 49.72) | 44.54 (42.30 – 46.95) | 0.22 |
| **VAT volume [cm^3^]** | | 420 (251 – 761.5) | 775.5 (297.75 – 1275) | 0.29 |
| **VAT mass [g]** | | 396 (237.5 – 718.5) | 731.5 (291,75 – 1203.25) | 0.24 |
| **HbA1c [mmol/l]** | | 4.9 (4.75 – 5.15) | 5.1 (4.9 – 5.1) | 0.42 |
| **OGTT** | **glc 0’ [μIU/l]** | 97 (90.75 – 98.75) | 94 (91.5 – 95.5) | 0.25 |
|  | **AUC glc** | 183 (171.25 – 199.5) | 175 (166.5 – 193.5) | 0.45 |
|  | **ins 0’ [μIU/l]** | 7.63 (6.27 – 11.82) | 8.827 (7.39 – 10.91) | 0.48 |
|  | **AUC ins** | 22.38 (14.65 – 33.40) | 26.115 (21.91 – 51.91) | 0.19 |
| **Cholesterol [mg/dl]** | | 155 (135 – 177.5) | 174 (148.25 – 182) | 0.40 |
| **LDL-cholesterol [mg/dl]** | | 77.6 (65.9 – 111.5) | 101.5 (77.25 – 106.5) | 0.41 |
| **HDL-cholesterol [mg/dl]** | | 52 (39 – 73) | 57.5 (47.5 – 72.5) | 0.62 |
| **Triglycerides [mg/dl]** | | 86 (46.5 – 110) | 83 (46.25 – 108.5) | 0.98 |
| **CRP [mg/l]** | | 0.69 (0.255 – 2.65) | 0.74 (0.41 – 1.09) | 0.93 |
| **Creatinine [mg/dl]** | | 1 (0.9 – 1.075) | 0.91 (0.9 – 1.02) | 0.56 |
| **ALT [IU/l]** | | 21.8 (16.25 – 26.55) | 17.75 (15.65 – 28.3) | 0.54 |
| **AST [IU/l]** | | 20.4 (18.75 – 25.35) | 23.8 (18.25 – 30.95) | 0.66 |
| **TSH [μIU/ml]** | | 1.93 (1.45 – 3.19) | 1.87 (1.36 – 2.53) | 0.51 |

BAT – brown adipose tissue; BATpos – group possessing BAT; BATneg – group lacking BAT; % AT – percentage of adipose tissue in total body mass; % SMM - percentage of skeletal muscle in total body mass; VAT – visceral adipose tissue; OGTT – oral glucose tolerance test; glc 0’ – fasting glucose concentration; ins 0’ – fasting insulin level; AUC – area under the curve; HbA1c – glycated hemoglobin; CRP – C-reactive protein; ALT – alanine transaminase; AST – aspartate transaminase; TSH – thyrotropic hormone.
